# Hydroa Vacciniforme-like Lymphoproliferative disorder in an adult invades the liver and bone marrow with clear pathological evidence: a case report and literature review

**DOI:** 10.1186/s12879-020-05697-x

**Published:** 2021-01-06

**Authors:** Xiankun Wang, Peng Wang, Aibin Wang, Yanli Xu, Lin Wang, Zhihai Chen

**Affiliations:** 1 Center of Infectious Diseases, Beijing Ditan Hospital, Capital Medical University, 8 Jingshun East Street, Chaoyang District, Beijing, 100015 China; 2 Department of Pathology, Beijing Ditan Hospital, Capital Medical University, Beijing, China

**Keywords:** Hydroa Vacciniforme-like Lymphoproliferative disorder, Case report

## Abstract

**Background:**

Hydroa Vacciniforme-like Lymphoproliferative Disorder (HV-LPD) is the name given to a group of Epstein-Barr virus (EBV)-associated diseases. It resembles hydroa vacciniforme (HV), the rarest form of photosensitivity, and is a T-cell disorder associated with an Epstein-Barr virus infection. The majority of diagnosed cases occur in East Asia and South America. It is rare in the United States and Europe. Multiple studies have revealed the clinical manifestation of an enlarged liver, but no gold standard such as pathology has yet supported this as a clinical sign of HV-LPD.

**Case presentation:**

Here, we report a case of a 34-year-old Asian female with definite liver invasion. The patient had complained of a recurring facial rash for many years**.** The patient was admitted to the hospital because of an enlarged liver. After hospitalization, she was given an EB virus nucleic acid test. The EB virus nucleic acid test was positive, and pathological examination suggested that HV-LPD had invaded the skin, bone marrow, and liver. After being given antiviral treatment, the patient’s symptoms were mitigated.

**Conclusions:**

Our case confirms the liver damage was caused by HV-LPD and the effectiveness of antiviral treatment.

## Background

Hydroa vacciniforme-like lymphoproliferative disorder (HV-LPD) is a name given to a group of Epstein-Barr virus (EBV)-associated diseases. It resembles hydroa vacciniforme (HV), the rarest form of photosensitivity, and is associated with an Epstein-Barr virus infection. It is a primarily cutaneous form of EBV and encompasses the lesions previously referred to as HV and HV-like lymphoma (HVLL) [1]. All the T/NK-cell-EBV-associated diseases occur with higher frequency in Asians, and indigenous populations from Central and South America and Mexico. They are rare in the United States and Europe. In 2008, HVLL was listed as one of the Epstein-Barr virus (EBV)-positive lymphoproliferative disorders of childhood in the WHO Classification of Tumors of Haematopoietic and Lymphoid Tissues [2]. In 2016, WHO subsumed HVLL into Hydroa Vacciniforme-like Lymphoproliferative Disorder (HV-LPD) [3]. In the current study, we report on an adult patient with HV-LPD.

## Case presentation

The Ethics Committee of Beijing Ditan Hospital, Capital Medical University gave approval for this study and informed written consent was obtained from the patient. A 34-year-old Asian female had complained of a recurring facial rash for more than 24 years, recurring fever for more than 4 years, and an enlarged liver and spleen for 3 months. The patient had developed a facial rash more than 24 years ago, when she was less than 10 years old, and the rash fell off after the formation of a scab. The rash re-occurred after sun exposure 22 years ago and 19 years ago, and the rash healed itself within 1 week both times. Four years ago, the patient again had a facial rash, with no obvious cause, accompanied by afternoon fever. Her body temperature was approximately 37.5 °C, accompanied by fatigue and night sweats. The patient went to the Baoding Infectious Disease Hospital where she was given an EB virus nucleic acid test and it was found that the EB virus nucleic acid test was positive (10^5^ to 10^6^ copies/ml) and the spleen was enlarged. The above symptoms were not mitigated after treatment with interferon, so she went to You’an Hospital for treatment, where she was treated with Acyclovir, and her symptoms were relieved, but the condition was not followed up. Three years ago, in the early and mid-stages of pregnancy, the patient developed a rash, which worsened in the third trimester. The patient did not see a doctor. After cesarean section delivery of her child, the patient developed a fever. She took Ganciclovir but the EBV nucleic acid remained positive (10^5^ to 10^6^ copies/ml). She was treated with Acyclovir and the symptoms were relieved. Three months ago, a physical examination revealed the presence of an enlarged liver and spleen, leading the patient to visit the Ditan Hospital for treatment. Since the onset of the disease, the patient has exhibited symptoms of fatigue, normal appetite, and no significant change in body weight. Her medical and personal history includes a caesarean section 2 years ago followed by normal menstrual cycles, no regular medication, rare alcohol intake, and no history of traveling abroad or recent blood transfusions. She denied having family members with similar diseases. A physical examination of the patient revealed a body temperature of 38.2 °C, blister scars distributed on the face, and enlargement of the liver and spleen, which could be felt under the ribs. All other examined parameters were normal.

**Fig. 1:**
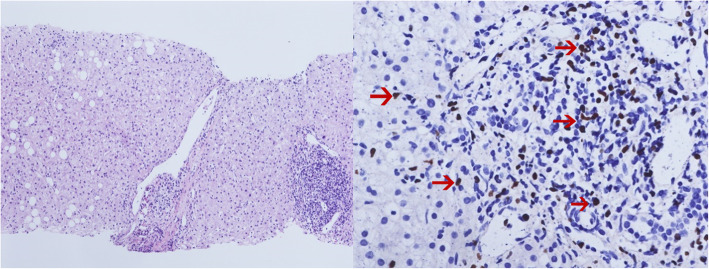
The liver biopsy of the patient. Obvious periportal lymphocytic infiltration and steatosis were observed. It mimicked a common hepatic virus infection (Right, H&E stain at magnification of 40). The EBER ISH stain showed the lymphocytic cells were mostly positive, indicating most were tumor cells due to the lymphocyte homing mechanism. (Left, EBER ISH at magnification of 400)

**Fig. 2:**
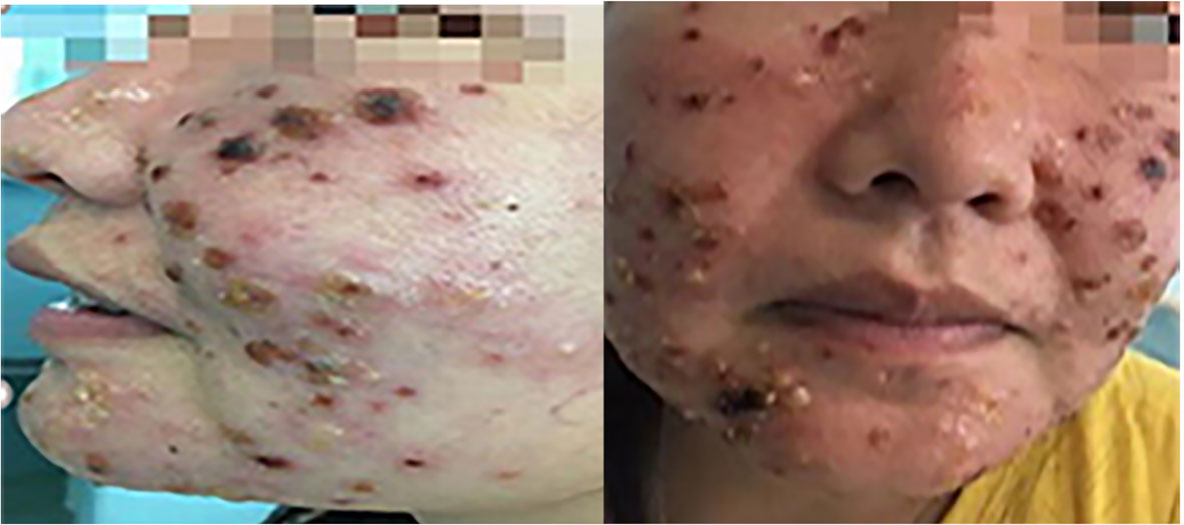
Incidences of erythema, pimples, and blisters appearing on the light-exposed parts of the patient’s face

**Fig. 3:**
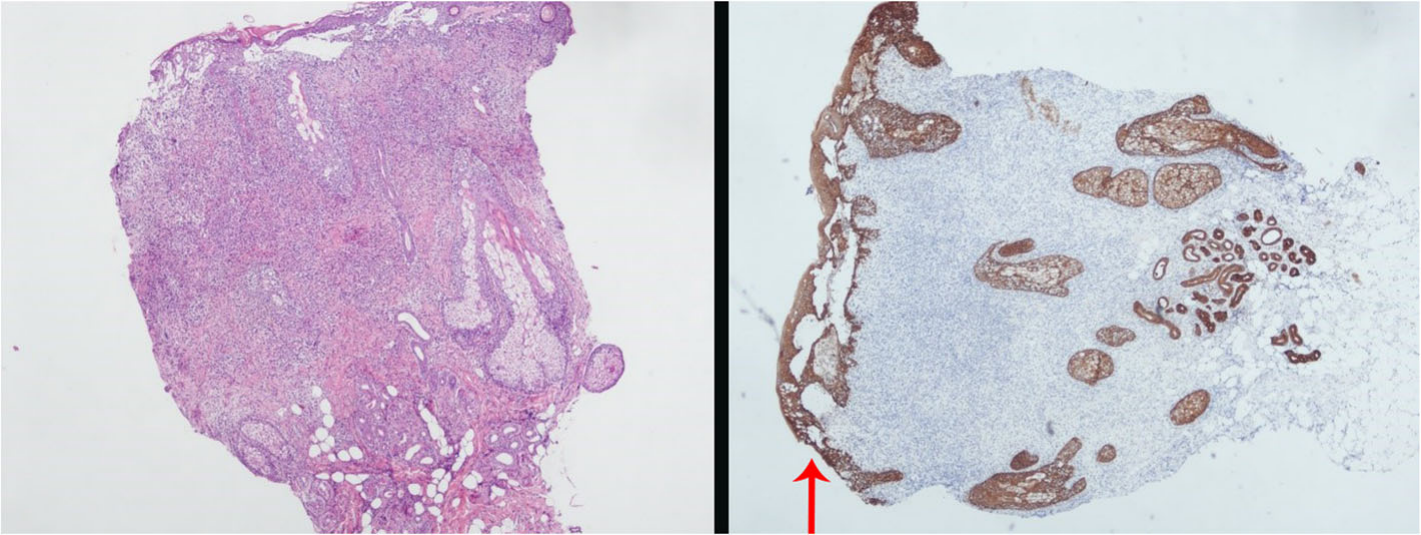
Skin biopsy from the patient’s face. The massive atypical T lymphocyte not only infiltrated the dermis, but also the epidermis and occasionally the subcutaneous adipose (Right, H&E stain at magnification of 40). AE1/AE3 immunohistochemistry stain of the same skin biopsy. The skin and cutaneous adnexae were observed to be completely involved by the tumor cells (Left, IHC stain at magnification of 40)

**Fig. 4:**
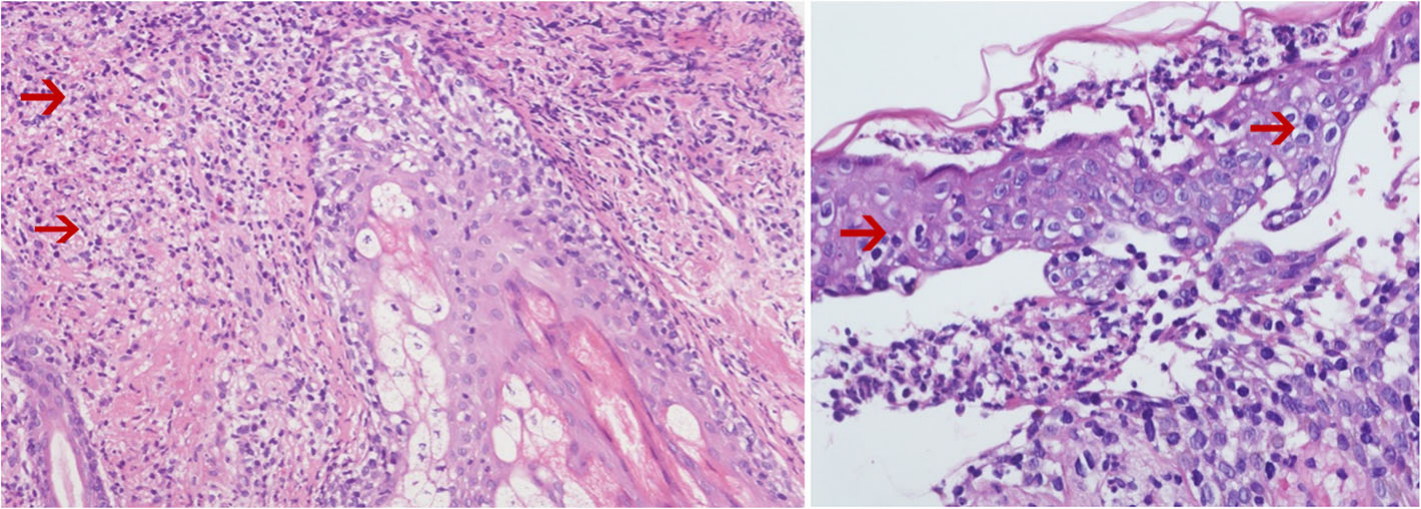
The tumor cells consisted of a diverse set of T cells with mixed reactive eosinophilic cells. These cells invaded the sebaceous and sudoriferous glands (Right, H&E stain at magnification of 200). The figure on the left shows the tumor cells invaded the epidermis and the lesions made it vacciniforme-like (Left, H&E stain at magnification of 400)

**Fig. 5:**
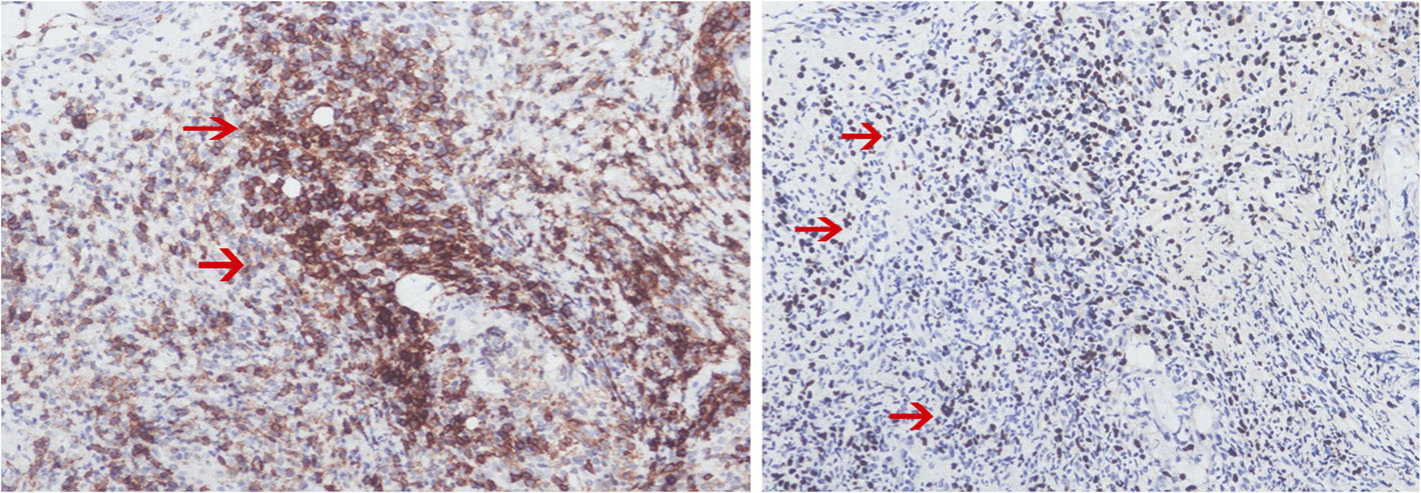
The tumor cells were predominantly CD4^+^ T cells with the T-killer phenotype (Right, IHC CD4 stain at magnification of 200). The EBER ISH stain showed the majority of the tumor cells were positive, which was fundamental to make the final diagnosis (Left, EBER ISH at magnification of 200)

**Fig. 6:**
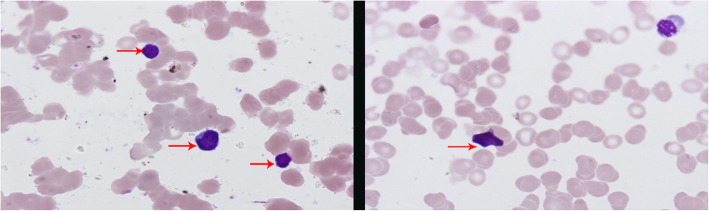
The bone marrow smear of the same patient (Diff-Quik stain, 1000x magnification). The immature or bizarre lymphocytes were observed in numerous fields. The morphology of the lymphocytes was consistent with T lymphoma. The bone marrow involvement was similar to the liver involvement. Further flow cytometry analysis confirmed the portion of tumor cells

Upon admission to the hospital (2019-7-1), the patient had a white blood cell count of 2.65 × 10^9^/L, and a neutrophil count of 1.16 × 10^9^/L. Liver function tests showed the alanine aminotransferase (ALT) was 40.8 U/L, and aspartate transaminase (AST) was 54.6 U/L. The EB virus nucleic acid quantification was significantly higher than normal at 3.33 × 10^7^ copies/ml. The antinuclear antibody karyotype exhibited a nuclear homogeneity with a ratio of 1:1000. The serum ferritin detection value was 32.20 ng/ml. T cell subset detection showed that T lymphocytes accounted for 97.34% of the lymphocytes and CD4^+^ T lymphocytes accounted for 24.25% of the lymphocytes. The lymphocyte concentrations were 340 cells/ul and 16 cells/ul for CD4^+^ T cells and natural killer cells, respectively. The CD4^+^ T lymphocytes accounted for 0.95 of the CD8^+^ T lymphocytes. The above laboratory results are shown in Table 1. A lymph node ultrasound showed several lymph nodes which were medullary clearing in the bilateral neck (the larger one on the left side was 14 × 5 mm, and the larger one on the right side was 13 × 4 mm), in the bilateral axilla (the larger one on the left was 10 × 4 mm, and the one on the right was 8 × 5 mm), and in the bilateral groin area (the larger one on the left side was 9 × 4 mm, and the larger one on the right side was 10 × 3 mm). Abdominal enhanced computed tomography showed enlargement of both the liver and spleen and thickening of the portal vein. Abdominal ultrasound also showed liver enlargement (the left lobe thickness of the liver was 78 mm, the long diameter was 115 mm, and the right lobe was 163 mm), diffuse liver lesions, an enlarged spleen (spleen length was 217 mm), and a rough gallbladder wall. Chest X-ray and uterine attachment ultrasound showed no abnormalities. Despite treatment with Acyclovir, the patient’s EBV nucleic acid load did not decrease significantly. Thus, the antiviral treatment regimen was changed to Ganciclovir combined with Sodium Phosphate. The liver function was abnormal, with enlargement, suggesting that the HV-LPD involved the liver tissue. Thus, we completed a liver biopsy (Fig. 1). The patient had a facial rash (Fig. 2) that had recently gradually worsened, and we treated her with oral Doxycycline and completed a skin biopsy. The biopsy results demonstrated a deeply stained heterotypic lymphocyte nucleus, which is consistent with HV-LPD (Figs. 3, 4 and 5). We also completed a bone marrow puncture due to the patient developing a decrease in the number of leukocytes and showing active myeloproliferative neoplasm and suspected lymphoma involvement (Fig. 6). Combined with the patient’s history, physical signs, and examination, the patient was diagnosed with HV-LPD. After treatment with antiviral therapy, the patient’s rash subsided and no fever was reported. The EBV nucleic acid in the whole blood was at an almost normal level, the viral nucleic acid in the serum was at a normal level, and the liver function returned to the normal level (2019-7-31). The above laboratory results are shown in Table 1.

**Table 1:** Changes in laboratory parameters during the hospitalization

	2019-7-1	2019-7-16	2019-7-31	Reference range
WBC (10^9^/L)	2.65	3.22	2.47	4 ~ 10
NEU(10^9^/L)	1.16	1.99	1.59	2 ~ 8
LYM (10^9^/L)	1.25	1.02	0.62	1 ~ 5
MON (10^9^/L)	0.05	0.18	0.25	0.2 ~ 0.8
EO (10^9^/L)	0.01	0.02	0	0.02 ~ 0.5
BASO (10^9^/L)	0.18	0.01	0.01	0 ~ 0.1
Hb (g/L)	112	27.76	116	110 ~ 150
PLT (10^9^/L)	136	191.5	151.5	100 ~ 300
ALT (U/L)	40.8	18.9	23.6	7 ~ 40
AST (U/L)	54.6	25.2	38.6	13 ~ 35
TBIL (μmol/L)	10.2	9.3	10	0 ~ 18.8
DBIL (μmol/L)	4.8	3.1	3.5	0 ~ 6.8
TP (g/L)	70.2	71.2	70.8	65 ~ 85
ALB (g/L)	39.4	41.1	40.9	40 ~ 55
ALP (U/L)	148	135.2	138.2	35 ~ 100
GGT (U/L)	9059	9891	8801	7 ~ 45
TBA (μmol/L)	4	3.5	4.4	0 ~ 10
sCr (μmol/L)	175	297	177	41 ~ 73
LDH (U/L)	282.6	202.4	209.3	120 ~ 250
The antinuclear antibody	1:1000	NA	NA	< 1:100
Ferritin (ng/ml)	32.2	NA	NA	11 ~ 306.8
T lymphocytes/ lymphocytes (%)	93.34	NA	NA	56 ~ 85
T lymphocytes (cells/UL)	1365	NA	NA	1027 ~ 2086
CD4+ T lymphocytes/ T lymphocytes (%)	25.56	NA	NA	30 ~ 54
CD4+ T lymphocytes (cells/UL)	359	NA	NA	706 ~ 1125
CD4+ T lymphocytes/ T lymphocytes (%)	24.25	NA	NA	15 ~ 34
CD8+ T lymphocytes (cells/UL)	340	NA	NA	320 ~ 1250
CD4+ T lymphocytes/ CD8+ T lymphocytes (%)	0.95	NA	NA	1 ~ 2
Natural killer cells (cells/UL)	16	NA	NA	90 ~ 590
B lymphocytes (cells/UL)	18	NA	NA	90 ~ 660
EB virus nucleic acid^a^ (copies/ml)	3.33 × 10^7^	2.92 × 10^7^	4.55 × 10^4^	4 × 10^2^
EB virus nucleic acid^b^ (copies/ml)	NA	NA	< 4 × 10^2^	4 × 10^2^

*WBC* White blood cell, *NEU* Neutrophil, *LYM* Lymphocyte, *MON* Monocyte, *EO* Eosinophilic cell, *BASO* Basophil, *Hb* Hemoglobin, *PLT* Platelet count, *ALT* Alanine aminotransferase, *AST* Aspartate aminotransferase, *TBIL* Total bilirubin, *DBIL* Direct bilirubin, *TP* Total protein, *ALB* Albumin, *ALP* Alkaline phosphatase, *GGT* Gamma glutamine transferase, *TBA* Total biliary acid, *sCr* Serum creatinine, *LDH* Lactate dehydrogenase, *NA* Not applicable

EB virus nucleic acid^a^ means EB virus nucleic acid in blood

EB virus nucleic acid^b^ means EB virus nucleic acid in serum

## Discussion and conclusion

HV-LPD is a cutaneous form of the Epstein-Barr virus-positive T/NK lymphoproliferative disease that is ethnically specific and puts patients at risk of systemic lymphoma [4]. The majority of diagnosed cases occur in Asia and South America [5]. It is rare in Europe and the United States. The disease is more common in children and adolescents, with a small number of cases of adults [6] and the elderly reported [5]. It is more common in females than in males. The patient we reported here became infected at an early age, less than 10 years old, and the disease was aggravated after childbirth. There are no current reports that fertility is an attenuated or aggravating factor for the disease.

The disease includes both local and systemic clinical manifestations. Local manifestations are characterized by whole-body light exposure sites, such as facial and hand skin lesions, which are characterized by recurring papules, blisters, ulcers, and gradual crusting. After the skin lesions heal, acne-like marks are left behind [7]. There may be facial and periorbital edema, and conjunctival and corneal ulcers may occur in the eye [8]. There is literature suggesting that the severity of the rash in adults is milder than in children [9]. Asian patients are more likely to be associated with allergy to mosquito bites [7]. Some patients develop systemic symptoms as the disease progresses, including general malaise, fever, weight loss, enlarged liver and spleen, enlarged lymph nodes, and anemia. Hematological examination reveals reduced hemoglobin, low blood platelet count, decreased hematocrit, and elevated lactate dehydrogenase [7, 10]. Typically, most of the bone marrow smears are normal. The patient we reported had typical clinical manifestations of skin damage, fever, and enlarged liver and spleen, and uncommon symptoms such as a decrease in the number of leukocytes, elevated transaminases, and elevated bilirubin, but no increase in lactate dehydrogenase, no low blood platelet count, or anemia. The patient’s positive antinuclear and anti-neutrophil cytoplasmic antibodies are considered to be related to the Epstein-Barr virus and autoimmune activation. The T cell subsets in the blood of the patient we reported showed a decrease in the number of CD4^+^ T cells, a decrease in CD4^+^/CD8^+^, a decrease in the number of NK cells, and a decrease in B lymphocytes, indicating that the patient’s cellular and humoral immunity was disordered. These phenomena are rarely reported in HV-LPD, illustrating the novelty of the information provided in this case study.

Most skin biopsies show epidermal necrosis, epidermal sponge edema, reticular degeneration, massive lymphocyte infiltration in the dermis, and occasional subcutaneous tissue involvement. Lymphocytes are distributed around the blood vessels and destroy the blood vessels. Proliferating lymphocytes are mostly small to medium-sized with unobvious nuclear heterogeneity [11]. The immunohistochemical feature of most patients is the T lymphocyte phenotype, which is positive for CD4 or CD8 and positive for CD2, CD3, CD30, and CD45Ro. A small proportion of T cells show the NK cell phenotype (CD56 positive) [11]. The skin biopsy we reported in this patient showed a large number of atypical lymphocytic infiltrations in the dermis with significant heterogeneity, which is rarely reported. At the same time, the patient’s immunological examination presented the CD4^+^ T lymphocyte phenotype. Liver enlargement has been repeatedly mentioned in the literature reports to date, but the cause of the swelling is not confirmed by the current gold standard pathology. The patient we reported had a liver puncture, and the pathological findings suggest that the patient’s liver enlargement was indeed associated with the disease, not autoimmune liver disease. EBV-encoded RNA (EBER) (+) was found in the pathological tissues of the skin, which also confirmed that the disease was associated with the EB virus infection. EBV usually is a long-term latent infection in B lymphocytes, but in HV-LPD, the virus infects T lymphocytes/natural killer cells, leading to clonal proliferation. The BamHI-Z leftward reading frame-1 (BZLF-1) protein is a product of the immediate early genes of EBV, which induces EBV reactivation and produces cleavage-cycle-associated antigens that stimulate the immune response of cytotoxic T lymphocytes [12].

Current medical reports suggest that immunomodulation and antiviral therapy have a better therapeutic effect on the disease. Zhang et al. gave HVLL patients 1.5 million U interferon injections every other day, which led to significant improvement in patient symptoms within 6 months, including disappearing facial edema and fever [13]. Quintanilla-Martinez et al. treated HVLL patients with immunosuppressive agents, such as thalidomide, glucocorticoids, hydroxychloroquine, etc., causing the symptoms to be alleviated, and multiple biopsies showed a decrease in the number of EBV-positive cells in the lesions [10]. Lysell et al. examined 4 cases of HV children with oral acyclovir for 2 to 4 weeks, and the symptoms of skin lesions and fatigue showed improvement [14]. At present, allogeneic hematopoietic stem cell transplantation also shows good efficacy in the treatment of this disease. El-Mallawany et al. performed EBV-positive donor allogeneic hematopoietic stem cell transplantation in a patient with chemotherapeutic resistance to HVLL and concurrent donor-derived EBV-specific cytotoxic T lymphocyte infusion immunotherapy, with long-term remission. At the year follow-up, the T cell receptor (TCR) gene rearrangement and EBER were negative [15]. For HV-LPD, chemotherapy and radiotherapy are not recommended as routine treatments. Kimura et al. reported that patients receiving chemotherapy were only temporarily relieved and could not maintain long-term complete remission [16]. In general, HV and HV-LPD are difficult to treat and conservative treatment should be used for the first-line treatment of HV, such as immunomodulatory therapy (glucocorticoids, thalidomide, hydroxychloroquine, cyclosporine, etc.). For HV-LPD, chemotherapy can only temporarily relieve the disease and cannot maintain long-term complete remission. Thus, the overall efficacy of chemotherapy is not high and chemotherapy should not be used as a routine treatment. Early hematopoietic stem cell transplantation has shown promising results, but treatment indications and standard therapy have not been determined.

Guo et al. showed that an EBV-DNA high nucleic acid load, elevated lactic dehydrogenase, cytopenia, and multiple organ injury were poor independent prognostic factors, and TCR gene rearrangement could be used as a reference for evaluating the progression of the HV [7]. Cohen et al. proposed that ethnicity is a serious influencer of the severity of the disease, suggesting that Caucasians show milder symptoms than other races and the disease progresses more slowly [17]. Miyake et al. considered that expression of the EBV product BZLF-1 mRNA is a death-related risk factor for univariate analysis of EBV-associated lymphoproliferative disorders [12]. The patient we reported has multiple organ damage, elevated liver transaminases, and high viral loads of Epstein-Barr virus nucleic acid, which indicates a poor long-term prognosis. Therefore, if a patient’s conditions permit the treatment, we recommend that patients undergo allogeneic hematopoietic stem cell transplantation as soon as possible.

The clinical manifestations, histopathology, genetics, and cytology of this disease are diverse and complex, and the pathogenesis of EBV remains unclear. Previous literature suggests the occurrence of patient liver enlargement, but there is no pathological gold standard to support that liver enlargement is directly related to the disease. However, the liver biopsy in this reported case suggests that liver enlargement is caused by this disease, which may reveal part of the pathogenesis of the disease. The total number of cases of the disease reported in the literature is small, and the literature also lacks long-term follow-up data. Thus, more case data should be accumulated for the study of the etiology, malignant transformation, and prognosis-related risk factors of the disease to provide direction for clinical diagnosis and treatment.

## Data Availability

The datasets used and analyzed in this study are available from the corresponding author on request.

## Abbreviations

HV-LPD: Hydroa Vacciniforme-like Lymphoproliferative Disorder
EBV: Epstein-Barr virus
HV: Hydroa vacciniforme
HVLL: Hydroa vacciniforme-like lymphoma
ALT: Alanine aminotransferase
AST: Aspartate transaminase
TCR: T cell receptor
EBER: EBV-encoded RNA
BZLF-1: BamHI-Z leftward reading frame-1
